# Rapid Qualitative Urinary Tract Infection Pathogen Identification by SeptiFast^®^ Real-Time PCR

**DOI:** 10.1371/journal.pone.0017146

**Published:** 2011-02-16

**Authors:** Lutz E. Lehmann, Stefan Hauser, Thomas Malinka, Sven Klaschik, Stefan U. Weber, Jens-Christian Schewe, Frank Stüber, Malte Book

**Affiliations:** 1 University Department of Anaesthesiology and Pain Therapy, Inselspital, Bern University, Bern, Switzerland; 2 Clinic and Policlinic for Urology, University Hospital Bonn, Bonn, Germany; 3 Kantonsspital Aarau AG, Department of Surgery, Aarau, Switzerland; 4 University Department of Anaesthesiology and Intensive Care Medicine, University Hospital Bonn, Bonn, Germany; Charité-University Medicine Berlin, Germany

## Abstract

**Background:**

Urinary tract infections (UTI) are frequent in outpatients. Fast pathogen identification is mandatory for shortening the time of discomfort and preventing serious complications. Urine culture needs up to 48 hours until pathogen identification. Consequently, the initial antibiotic regimen is empirical.

**Aim:**

To evaluate the feasibility of qualitative urine pathogen identification by a commercially available real-time PCR blood pathogen test (SeptiFast^®^) and to compare the results with dipslide and microbiological culture.

**Design of study:**

Pilot study with prospectively collected urine samples.

**Setting:**

University hospital.

**Methods:**

82 prospectively collected urine samples from 81 patients with suspected UTI were included. Dipslide urine culture was followed by microbiological pathogen identification in dipslide positive samples. In parallel, qualitative DNA based pathogen identification (SeptiFast^®^) was performed in all samples.

**Results:**

61 samples were SeptiFast^®^ positive, whereas 67 samples were dipslide culture positive. The inter-methodological concordance of positive and negative findings in the gram+, gram- and fungi sector was 371/410 (90%), 477/492 (97%) and 238/246 (97%), respectively. Sensitivity and specificity of the SeptiFast^®^ test for the detection of an infection was 0.82 and 0.60, respectively. SeptiFast^®^ pathogen identifications were available at least 43 hours prior to culture results.

**Conclusion:**

The SeptiFast^®^ platform identified bacterial DNA in urine specimens considerably faster compared to conventional culture. For UTI diagnosis sensitivity and specificity is limited by its present qualitative setup which does not allow pathogen quantification. Future quantitative assays may hold promise for PCR based UTI pathogen identification as a supplementation of conventional culture methods.

## Introduction

The initial treatment of urinary tract infections (UTI) is mostly empirical. The immediately started therapy targets to avoid serious complications such as UTI triggered urosepsis and shortening the time of patients' discomfort. The diagnosis of UTI is based on three criteria: i) Clinical symptoms ii) Detection of signs of infection in the urine iii) Detection and identification of bacteria in the urine. The gold standard for pathogen identification is the urine culture by plated midstream urine [1]. The time need for preliminary results of the urine culture is at least 24 hours and final results are commonly available after 48 hours [2].

To date, molecular biology techniques such as real-time PCR are used to complement conventional culture methods, especially with regard to shortening the time to result [3], [4]. In the diagnosis of UTI applied real-time PCR methods are presently limited to the detection of single pathogens or the Gram status [5], [6]. Recently, we showed the principle feasibility of UTI pathogen identification by an in-house developed real-time PCR as a supplement for culture methods [7]. The main advantage in the use of real-time PCR techniques is the considerable saving of time.

A new multiplex real-time PCR test for the detection of 25 common blood stream pathogens (SeptiFast^®^, Roche Diagnostics GmbH, Penzberg, Germany) has been introduced recently [4]. An overlap of probe binding sites leads to the final discrimination of 20 common blood pathogens. The aim of this prospective pilot study was to investigate the feasibility of the quantitative detection and identification of urine pathogens in UTI patients by the commercially available SeptiFast^®^ test. Moreover, the SeptiFast^®^ results were compared with the findings of the conventional dipslide based pathogen detection and the results of the in-house PCR method. Although there is an overlap in the panels of frequent of UTI and blood stream infection pathogens it has to be pointed out that the SeptiFast^®^ panel was not developed or modified to detect UTI pathogens. Subsequently, there are relevant UTI pathogens such as Citrobacter which are not represented in this panel [8].

## Methods

### Patient cohort

**Figure 1:**
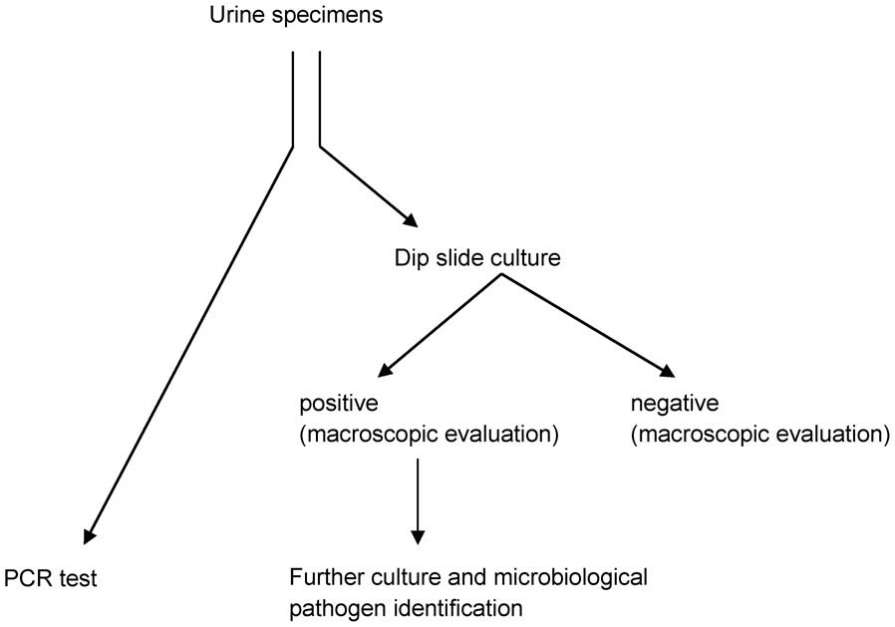
Workflow of the urine specimens to the different tests. Samples were classified as positive and count as one in case of mono or multiple infection.

The study was approved by the ethics committee of the medical faculty of the Rheinische Friedrich-Wilhels-University Bonn and complied with all relevant guidelines. The inclusion criteria were: Urological patients with age ≥18 years, suspected UTI, absence of enterovesical fistulas, and written informed consent. Patients with age <18 years, without written informed consent, enterovesical fistulas, urinary diversion or bladder augmentation were excluded. The routine course in case of suspected UTI consisted of: i) Physical examination by fellows or consultants of the Department of Urology. ii) Semi quantitative urine analysis by commercially available urine dipsticks (Combur-Test®, Roche Diagnostics, Mannheim, Germany) applied according to the manufacturer's recommendations. iii) Identification of pathogens by dipslide based culture method (Dip-Slide Cled/MacConkey/malt extract agar®, Oxoid, Wesel, Germany) applied according to the manufacturer's recommendations and subsequent microbiological pathogen identification. The arrangement of the samples is visualized in figure 1. Microbiological culture data of the patients were partially included in a previous report [7]. In 82 samples we compared the SeptiFast^®^ results with the previously published results detected with a in-house real-time PCR method [7].

### Urine sample preparation and conventional urine analysis

Urine sampling was performed according to local hospital guidelines for preventing artificial contamination. A sterile specimen holder with Cled-, MacConkey-agar, and malt extract agar (Dip-Slide Cled/MacConkey/malt extract agar, Oxoid, Wesel, Germany) was dunked in the urine sample and cultured for 24 hours at 36°C. In case of visible bacterial growth the sample was transferred to the Institute of Microbiology for pathogen identification. Analysis was done according to standards of the German Society of Medical Microbiology and Hygiene and the local hospital guidelines. A sample was regarded as a true positive infection when >10^5^ CFU/ml urine were detected [9]–[11].

### PCR sample preparation and PCR procedure

A 10ml midstream urine sample for PCR diagnostic was transferred on ice to the PCR laboratory. For complete accordance to the SeptiFast^®^ test guidelines the preparation of DNA and PCR testing was performed from 1 ml urine using the PCR Lysis Kit, the SeptiFast^®^ Prep Kit and the LightCycler SeptiFast^®^ Kit as described recently [4]. In brief, samples were mechanically lyzed and internal controls (IC) were included in each sample and in negative controls (NC). Manual extraction was performed to obtain a final extraction volume of 200 µl DNA. 50 µl of eluate were used for the subsequent real-time PCR amplification using the LightCycler 2.0 Instrument. Potential process contaminations were eliminated using uracil-N-glycosylase. DNA amplification targets conserved and variable parts of the internal transcribed sequence regions of bacteria and fungi [4]. Amplified variable parts of any amplification products were then hybridized to genus- or species-specific oligonucleotide probes and subjected to software-assisted temperature melting-peak (Tm) analysis using the Bacterial and Fungal Identification Software Package Version 3.0.4.28 (Roche Diagnostics GmbH, Penzberg, Germany) to reliably identify microorganisms. The SeptiFast^®^ pathogen panel is displayed in table 1. SeptiFast^®^ results were regarded as true negative only if included IC's are measured positive. Moreover, the assay was regarded valid only if the NC tested negative and the corresponding controls (reagent control and the IC of the NC) were detected within their assigned Tm ranges. According to recent study data time to report for the method's workflow is less than four hours and the analytical sensitivity of the assay ranges between 3 and 100 CFU/ml in whole blood depending on the individual microorganism [4]. Presently, no data concerning the analytical sensitivity in urine are available by the manufacturer.

**Table 1:** Pathogens included in the SeptiFast^®^ detection panel.

Gram positive	Gram negative	Fungi
*Staphylococcus aureus*	*Escherichia coli*	*Candida albicans*
*Coagulase negative staphylococci*	*Klebsiella pneumonia*	*Candida tropicana*
*Streptococcus pneumoniae*	*Serratia marcescens*	*Candida parapsilosis*
*Streptococcus spp.*	*Enterobacter*	*Candida krusei*
*Enterococcus faecium*	*Proteus mirabilis*	*Candida glabrata*
*Enterococcus faecalis*	*Pseudomonas aeruginosa*	*Aspergillus fumigatus*
	*Acinetobacter baumanii*	
	*Stenotrophomonas maltophilia*	

### Statistical Methods

For the calculation of sensitivity and specificity of SeptiFast^®^ test the microbiological culture and identification method was considered as the gold standard method. The comparison of both methods was performed by the calculation of the positive and negative predictive value and Cohen's kappa coefficient. On pathogen level the species specific overall concordance was calculated as the ratio of the sum of positive and negative identical results to the entire number of results. For the analysis of mono- or polymicrobial infections concordance was calculated as the sum of positive identical results to the entire number of results.

## Results

81 patients were enrolled in the study. One patient suffered from two consecutive UTI episodes which resulted in 82 samples from 81 patients. Table 2 shows the baseline characteristics of the patient group. Initial dipslide culture results were available 24 hours after specimen collection. Subsequent conventional pathogen identification for positive dipslide samples required additional 24 hours. In contrast, PCR results were available 4.5 hours after specimen collection. Of note, results of this pilot study were not used for therapeutic decisions.

**Table 2:** Patient's baseline characteristics.

	Number	Percent (%)
Patients	81	
Samples	82	
Female	43/81	53
Male	38/81	47
Mean Age (range)	49 (18–79)	
Clinical entity		
- Lower urinary tract infections	39/82	48
- Pyelonephritis	33/82	40
- Obstructive pyelonephritis	10/82	12

Real-time PCR detected 61 infections and 21 infection-negative samples, whereas, culture method identified 67 infections and 15 infection-negative results. Table 3 shows the distribution of positive and negative results obtained with both methods. *Citrobacter species* was identified in four samples by culture based methods as a co-pathogen in multiple infected samples. Since this pathogen is not part of the SeptiFast^®^ detection panel it was excluded from concordance analysis. This resulted in a sensitivity of 0.82 and a specificity of 0.60 for the detection of an infection by the SeptiFast^®^ test in this pilot study. The overall Cohen's kappa coefficient is 0.364 and the positive and negative predictive values are 0.9 and 0.43, respectively. The concordant *positive* and *negative* findings (overall concordance) of the two methods were calculated on group level as well as on species specific level. Table 4 shows the results of the gram positive pathogens. In this group lowest concordance was observed for *Coagulase-negative staphylococci* identification 65/82 (79%), whereas, highest concordance was calculated for *Streptococcus pneumoniae* 81/82 (99%). The overall concordance was 371/410 (90%). In the gram negative group (table 5) the pathogen concordance ranged between 77/82 (94%) and 81/82 (99%) with lowest concordance for *Escherichia coli* and an overall concordance of 477/492 (97%). Finally, in the fungi group (table 6) the overall concordance was 238/246 (97%) and varied between 75/82 (91%) and reached 82/82 (100%) for *Candida crusei*. Of note, in this group including the pathogens *Candida albicans*, *Candida glabrata*, and *Candida crusei* only 11 pathogens were detected in total. The limited concordance in the gram positive group is based on 15 *Coagulase-negative staphylococci* and *Streptococcus species* pathogens identified exclusively by culture method.

**Table 3:** Detection of infections in 82 samples.

		Microbiological culture (n = 82 samples)		
		Positive infection	Negative infection	∑
Real-time PCR SeptiFast^®^ test (n = 82 samples)	Positive infection	55	6	61
	Negative infection	12	9	21
	∑	67	15	82
Kappa coefficient				0.364
Positive predictive value				0.90
Negative predictive value				0.43

**Table 4:** Gram positive pathogens.

	Exclusively Microbiology positive	Exclusively SeptiFast^®^ positive	Microbiology and SeptiFast^®^ positive	Microbiology and SeptiFast^®^ negative	Concordance [%]
*Coagulase-negative staphylococci*	14	3	7	58	65/82 [79]
*Staphylococcus aureus*	1	2	2	77	79/82 [96]
*Streptococcus pneumoniae*	0	1	0	81	81/82 [99]
*Streptococcus spp*	6	2	1	73	74/82 [90]
*Enterococcus spp.*	4	6	6	66	72/82 [88]
Overall	25	14	16	355	371/410 [90]
Kappa coefficient					0.4
Positive predictive value					0.53
Negative predictive value					0.93

**Table 5:** Gram negative pathogens.

	Exclusively Microbiology positive	Exclusively SeptiFast^®^ positive	Microbiology and SeptiFast^®^ positive	Microbiology and SeptiFast^®^ negative	Concordance [%]
*Escherichia coli*	4	1	32	45	77/82 [94]
*Klebsiella pneumonia*	2	0	6	74	80/82 [98]
*Serratia marcescens*	0	1	0	81	81/82 [99]
*Enterobacter*	1	2	0	79	79/82 [96]
*Proteus mirabilis*	2	0	0	80	80/82 [98]
*Pseudomonas aeruginosa*	0	2	1	79	80/82 [98]
Overall	9	6	39	438	477/492 [97]
Kappa coefficient					0.82
Positive predictive value					0.87
Negative predictive value					0.98

**Table 6:** Fungi.

	Exclusively Microbiology positive	Exclusively SeptiFast^®^ positive	Microbiology and SeptiFast^®^ positive	Microbiology and SeptiFast^®^ negative	Concordance [%]
*Candida albicans*	2	5	2	73	75/82 [91]
*Candida glabrata*	0	1	0	81	81/82 [99]
*Candida crusei*	0	0	1	81	82/82 [100]
Overall	2	6	3	235	238/246 [97]
Kappa coefficient					0.41
Positive predictive value					0.33
Negative predictive value					0.99

The concordant *positive* pathogen detections in monoinfections were 33/43 (77%) separated in 7/14 (50%), 25/28 (89%) and 1/2 (50%) in Gram positive, Gram negative and funghi, respectively. The overall concordance within the group of polymicrobial infections was 23/47 (49%). In this group Gram positive, Gram negative and funghi showed concordance in 9/27 (33%), 14/20 (70%) and 2/3 (67%) of the pathogen detections, respectively.

Finally, we compared the SeptiFast^®^ results with results from a previously performed in-house PCR of the same collective [7]. Table 7 shows the pathogen detection in 82 samples conducted with the SeptiFast^®^ method and the in-house method. 51 positive results showed concordant findings between SeptiFast^®^ and the in-house method.

**Table 7:** Pathogen detection with SeptiFast^®^ and an in-house PCR method.

Pathogen	SeptiFast^®^ positive (n)	In-house PCR [4] positive (n)	Concordant positive (n)
*Coagulase-negative staphylococci*	10	14	4
*Staphylococcus aureus*	4	3	3
*Streptococcus pneumoniae*	1	n.a.*	n.a.*
*Streptococcus spp*	3	5	1
*Enterococcus*	12	5	4
*Escherichia coli*	33	31	30
*Klebsiella pneumonia*	6	7	6
*Serratia marcescens*	1	0	0
*Enterobacter spp.*	2	1	0
*Proteus mirabilis*	0	1	0
*Pseudomonas aeruginosa*	3	3	3
*Candida albicans*	7	n.a.*	n.a.*
*Candida glabrata*	1	n.a.*	n.a.*
*Candida crusei*	1	n.a.*	n.a.*
Pathogen negative	21	21	14

*not applicable because not in the detectable panel of the in-house PCR [4].

## Discussion

### Summary of main findings

The presented data indicate for the first time, that the real-time PCR based SeptiFast^®^ system is applicable in principle for the qualitative identification of urinary tract pathogens. The time saving of the PCR based results is about 43 hours compared to the culture based pathogen identification. Compared to the gold standard method sensitivity and specificity of the real-time PCR method was 0.82 and 0.60, respectively, even though this study was designed as the proof of feasibility and not as a presentation of a ready to use method.

### Strengths and limitations of the study

This pilot study shows that PCR methods might supplement urine culture based pathogen identifications in the future. The most important benefit is the faster pathogen identification which allows an earlier selective antimicrobial therapy. In this first study the authors decided to deploy the SeptiFast^®^ blood pathogen test in full accordance to its instruction to answer the question whether it is feasible in urine samples. In addition, the study was not designed to analyse the pathogen detection limit of UTI pathogens in urine. Therefore, the existing blood culture data might be seen as a substututional reference point of the analytical sensitivity. In summary, the real-time PCR method was used off-label in urine.

The most important difference between the blood and the urine compartment in the diagnosis of pathogens is the need for quantification in UTI diagnosis. In contrast, the qualitative detection of pathogens is sufficient for the diagnosis of blood stream infections.

The overall kappa coefficient indicated a very limited concordance between the two methods as well in the analysis of gram positive and gram negative bacteria and fungi. Moreover, the positive and negative predictive values in the different groups showed variable proportions of correctly diagnosed patients, respectively pathogens. However, this investigation was designed as a test of the methodological feasibility and not as the rating of a new method against the Goldstandard.

*Coagulase-negative staphylococci* and *Streptococcus spp.* were frequently detected by the culture method exclusively, which contributed to the SeptiFast^®^ sensitivity of 0.82. When turning off the manufacturer's given filter for these two pathogens they were detected by real-time PCR in further 35 samples. With the filter engaged these signals were not assessed as positive findings, because the PCR crossing points were too high and exceeded the SeptiFast^®^ detection window constituted for blood stream infections. This filter was implemented in the SeptiFast^®^ system for the reduction of false positive results due to contamination with cutaneous pathogens in the blood infection setting. It is important to mention that this filter is not validated for the detection of UTI pathogens in urine specimens. In the presented research setting a manual inspection of each PCR crossing point is feasible. However, prior to the potential future use of real-time PCR based tests the validation of a cut off value to discriminate infection from contamination is mandatory. It could be speculated that a modification of this implemented filter might increase the sensitivity by higher numbers of detected *coagulase-negative staphylococci* and *Streptococcus spp*.

An alternative method for pathogen concentration and subsequent increase of PCR sensitivity is the urine centrifugation prior to analysis. However, there was no concentration step in this study. It can be argued that this step might be beneficial to increase the overall sensitivity of this study.

The most striking results responsible for the limited level of specificity were 5 samples with exclusively SeptiFast^®^ positive *Candida albicans* and 10 samples with exclusively SeptiFast^®^ positive *Enterococcus* detection. Additionally, the cut off value applied in urine culture method detecting UTI is strongly suspicious to contribute considerably to the reported specificity of 0.60. Furthermore, the real-time PCR detection is limited by the fact that pathogens which are not included in the panel are undetectable and the number of included pathogens is restricted. In the present investigation four PCR undetected *Citrobacter species* findings, which are not included in the panel, demonstrated this limitation. However, the 8 most frequent outpatient UTI pathogens are included in the used SeptiFast^®^ panel [8], [12], [13].

Finally, there is the possibility to detect DNA of vital or degraded pathogens by real-time PCR in the urine of the patients. In the case of positive PCR finding but missing clinical UTI signs the relevance of the detected DNA is unclear. In this situation degraded DNA might just address a passed or subclinical infection. Up to now, the clearance of bacterial DNA from the urine is unclear. Moreover, evidence exists that a filtration of circulating DNA via the kidney is possible [14]. The contribution of this filtrated DNA to the total content of bacterial DNA in the urine remains to be established.

### Comparison with existing literature

The use of microbiological culture method is well established in the diagnosis of infectious diseases. However, the major drawback is the time consumption of this method. Therefore, initial antibiotic therapy in blood stream infections as well as in urinary tract infections is mostly empirical. Several investigations showed the disadvantages of delayed or inappropriate antimicrobial therapy, such as decreased survival rate in sepsis, or development of pathogen resistances [15]–[19]. Besides these serious complications, inappropriate UTI therapy extend patients time of discomfort.

In the field of blood stream infections real-time PCR based methods as SeptiFast^®^ were engineered to supplement culture based methods of pathogen identification and to reduce the time interval of calculated anti-infective therapies. In contrast to the qualitative PCR pathogen detection in blood stream infections, quantification is mandatory in the diagnosis of UTI to discriminate contamination from infection. The initial amount of pathogen DNA is determinable by the assessment of the PCR crossing point [20], [21]. Schabereiter-Gurtner and co workers reported for *Neisseria gonorrhoeae* real-time PCR sensitivity about 3 CFU [22]. Other groups reported the lower detection limit of real-time PCR be about 1 CFU per ml fluid or g tissue [23]–[25]. Consequently, future developments of commercial real-time PCR pathogen detection tools should include a quantification option.

The applied dipslide cultures were analysed by experienced urologic staff after 24 hour incubation. In case of visible growth microbiological routine diagnostic identified the pathogen 48 hours after specimen collection. This setup was chosen to fully comply with local hospital guidelines for routine UTI diagnosis. This might limit the study as plated midstream urine is commonly seen as the gold standard in urine culture diagnostics [1]. A further difference between the two methods is that culture methods exclusively detect viable and reproductive organisms, whereas PCR detect vital or dead pathogens as well as DNA fragments from degraded pathogens. Especially this fact might be useful in the monitoring of infections under antibiotic treatment. Such real-time PCR based diagnosis and disease monitoring tools are components of present treatment strategies in patients with viral infections [26]–[28].

To date, the acquisition of an antibiogram is possible exclusively by the culture technique. A real-time PCR triggered initial antibiotic therapy might be insufficient due to undetected antibiotic resistance. However, the routinely PCR detection of resistance genes as surrogate parameters for antibiotic resistance is practicable. Bacterial resistancy tests, such as the mecA-gene in *Staphylococcus aureus* and vanA/vanB in *Enterococcus species*, are today widely used in clinical routine approaches for characterisation of resistant strains [29]. Therefore, an expansion of the PCR based UTI pathogen identification by the detection of resistance genes might be a perspective to enhance UTI diagnosis as well.

The comparison of the SeptiFast^®^ method with a recently published in-house real-time PCR method with pathogen specific detection probes [7] showed concordant pathogen results in about 70% of the positive samples. In both real-time PCR based methods an adoption to a quantitative approach was not performed. The detection probes of both PCR methods are different. Suboptimal template binding of several probes due to the secondary or tertiary DNA structure might contribute to intermethodological differences. An important difference between both methods is that the SeptiFast^®^ approach was designed as a multiplex PCR reaction. In contrast, the alternative PCR approach was designed as parallel PCR reactions and each of them included specific probes for one pathogen. In the view of practicability the multiplex SeptiFast^®^ method simplifies the procedure enormously.

### Implications for future research or clinical practice

A precondition for the future routine use of this technique is the quantification of colony forming units for decision making regarding significant infection, colonization, and contamination. Since real-time PCR DNA quantification is well established in clinical and scientific applications further research should model the quantification of the colony forming units by initial amount of DNA. The development of a standardized commercially available PCR based test for the identification of UTI pathogens and common resistance genes seems feasible. Of note, the accurate definition of the test panel is mandatory for its clinical relevance. Potentially, a differentiation between in-patients and out-patients pathogen panels is useful. Because of its high technical pre-requisits the method could be used in departments which are close connected to a microbiological laboratory which has to be familiar with real-time PCR methods. However, the trend to shift the diagnostic tools to the patients' bedside is unbowed. The growing use of microbiological point of care diagnostic is linked to its simplification. As a vision the development of PCR devices which choose the program and perform the analysis automatically might be possible. This step would bring microbiological point of care diagnostic to the general practitioner and in case of UTI's possibly more important to patients in less developed countries. For the short term real-time PCR methods might supplement the Goldstandard culture technique in medical centers. The combination with established culture methods might decrease the fraction of patients initially treated with inadequate antimicrobial therapy. A potential use of this PCR method can be seen for patients under antibiotic therapy due to recurrent UTI's or patients with nephrostomy under chronic antibiotic therapy with UTI symptoms. In such cases negative microbiological cultures are common. One can speculate that real-time PCR might serve as a useful adjunct.
